# ACE inhibitory peptides in standard and fermented deer velvet: an in silico and in vitro investigation

**DOI:** 10.1186/s12906-019-2758-3

**Published:** 2019-12-05

**Authors:** Stephen R. Haines, Mark J. McCann, Anita J. Grosvenor, Ancy Thomas, Alasdair Noble, Stefan Clerens

**Affiliations:** 1 AgResearch Limited, Lincoln Research Centre, Private Bag 4749, Christchurch, 8140 New Zealand; 2 AgResearch Limited, Grasslands Research Centre, Private Bag 11008, Palmerston North, 4442 New Zealand

**Keywords:** Deer velvet antler, Bioactive peptides, Simulated gastrointestinal digestion, ACE inhibitor peptides, In silico analysis, In vitro analysis

## Abstract

**Background:**

The use of deer velvet antler (DVA) as a potent traditional medicine ingredient goes back for over 2000 years in Asia. Increasingly, though, DVA is being included as a high protein functional food ingredient in convenient, ready to consume products in Korea and China. As such, it is a potential source of endogenous bioactive peptides and of ‘cryptides’, i.e. bioactive peptides enzymatically released by endogenous proteases, by processing and/or by gastrointestinal digestion. Fermentation is an example of a processing step known to release bioactive peptides from food proteins. In this study, we aimed to identify in silico bioactive peptides and cryptides in DVA, before and after fermentation, and subsequently to validate the major predicted bioactivity by in vitro analysis.

**Methods:**

Peptides that were either free or located within proteins were identified in the DVA samples by liquid chromatography-tandem mass spectrometry (LC-MS/MS) followed by database searching. Bioactive peptides and cryptides were identified in silico by sequence matching against a database of known bioactive peptides. Angiotensin-converting enzyme (ACE) inhibitory activity was measured by a colorimetric method.

**Results:**

Three free bioactive peptides (LVVYPW, LVVYPWTQ and VVYPWTQ) were solely found in fermented DVA, the latter two of which are known ACE inhibitors. However matches to multiple ACE inhibitor cryptides were obtained within protein and peptide sequences of both unfermented and fermented DVA. In vitro analysis showed that the ACE inhibitory activity of DVA was more pronounced in the fermented sample, but both unfermented and fermented DVA had similar activity following release of cryptides by simulated gastrointestinal digestion.

**Conclusions:**

DVA contains multiple ACE inhibitory peptide sequences that may be released by fermentation or following oral consumption, and which may provide a health benefit through positive effects on the cardiovascular system. The study illustrates the power of in silico combined with in vitro methods for analysis of the effects of processing on bioactive peptides in complex functional ingredients like DVA.

## Background

Deer velvet antler (DVA) has been used as a medicinal ingredient in Asia for over 2000 years, and is regarded as one of the most powerful animal-based remedies in the Chinese Pharmacopoeia. In addition, DVA is being included as a Healthy Functional Food ingredient in convenient consumer-ready forms such as drinks, health bars, chewing gum and probiotic yoghurt. This new usage is increasing so rapidly that by 2020 at least half of the DVA exported from New Zealand to South Korea and China will be utilised in such products (unpublished observations, C Stevenson, Deer Industry New Zealand). A range of biological activities of deer velvet has been demonstrated in rodent models, including anti-aging [1], anti-infective [2] and anti-inflammatory effects [3], protection against liver damage [4], haematopoietic activity [5], acceleration of wound healing [6], and reduction of grade and metastasis of azoxymethane-induced colon cancer [7].

Proteins and peptides constitute the major proportion of organic matter in DVA, and are likely responsible for many of its biological activities. The proteomics of DVA has been well described [8–10], but less is understood about its bioactive peptides. DVA is known to contain a range of tissue growth factors [11], and a 32-amino acid peptide has been shown to have anti-osteoporotic [12], wound healing [6] and immunomodulatory [13] activities. However, to the best of our knowledge, no global characterisation of the bioactive peptides in DVA has yet been published. A significant knowledge gap thus exists that reduces opportunities for exploitation of DVA as a functional food ingredient.

Considerable interest exists in the use of sequence information to predict the presence of bioactive peptides encoded in proteins from food and other natural sources, stimulated by the availability of searchable databases of biologically active peptide sequences. Multiple reports have appeared recently regarding the use of such databases to predict bioactivity that can potentially be released by proteolysis of food proteins, for example meat [14], milk [15] and plant proteins [16]. Some of these studies have compared proteins from different sources, and have determined which contain the greater frequencies of bioactive fragments. Typically, though, in silico digestion has been performed to predict the fragments that might be released from isolated source proteins, rather than to compare real mixtures of food peptides and protein.

In the present work, we instead used LC-MS/MS to first identify the sequences of peptides and proteins that were extractable from DVA and fermented DVA (FDVA), as described above. We then performed in silico analysis to compare the frequency of matches in the two samples to bioactive sequences in the BIOPEP, PeptideDB and APD2 databases, combined with a number of sequences derived from the scientific literature.

The peptides identified in each sample were also subjected to in silico simulated gastric digestion to predict whether or not bioactive sequences would be expected to survive (or be released by) exposure to pepsin following oral consumption.

Recently, in silico analysis has emerged as a valuable tool in the investigation of cryptides (i.e. bioactive peptides ‘encrypted’ within protein sequences) in complex mixtures derived from natural sources. For example, the online BIOPEP database and tools [17] have been used to predict bioactive peptides that could be released from meat, plant and dairy proteins [14, 16]. In most reported studies, in silico digestion has been performed to predict the fragments that might be released from isolated source proteins, rather than to compare real mixtures of food peptides and proteins. Our approach in the present work was to identify as many sequences as possible present in deer velvet either as free peptides or as tryptic fragments of intact proteins, using liquid chromatography-tandem mass spectrometry (LC-MS/MS) followed by database searching. We then predicted the presence of bioactive sequences in the complex mixtures by in silico matching against a database of bioactive peptides, and used in silico simulated gastric digestion to evaluate the potential effect of oral consumption on their release and survival. Since fermentation of foods is known to produce bioactive peptides, we compared a fermented deer velvet product [18] to the standard DVA powder from which it was produced, to determine if the predicted bioactivity of deer velvet was affected by hydrolysis of its proteins by bacterial enzymes in the fermentation mixture. Finally, having by this process identified inhibition of angiotensin-converting enzyme (ACE) as a major potential bioactivity of both DVA and fermented deer velvet antler (FDVA), we validated the results of the in silico analysis by determining the in vitro ACE-inhibitory activity of peptides extracted from both DVA and FDVA, before and after they were exposed in vitro to simulated gastrointestinal digestion.

## Materials and methods

### Materials

DVA and FDVA were prepared by UBBio Limited (Christchurch, New Zealand) using velvet antlers commercially sourced from red deer farmed in New Zealand. Protease inhibitor cocktail was from Roche (Indianapolis, IN, USA). Pepsin (1:2500, 150 units/mg, 600 units/mg protein; catalogue # P-7125) was sourced from Sigma (St. Louis, MO, USA) and pancreatin (from porcine pancreas; 4X USP-Units, minimum protease activity 1050 FIP-U/g; catalogue number A0585,0100) was from AppliChem (Darmstadt, Germany). All other chemicals were of analytical or proteomics reagent grade.

### Extraction

In order to ensure broad coverage of the deer velvet proteome and peptidome, DVA and FDVA were each extracted with five different buffers that provide complementary extraction of the proteins in deer velvet [8]. Samples were mixed for 23 h at 4 °C with either: (1) 1.2 M hydrochloric acid; (2) 2 M sodium hydroxide; (3) 100 mM *tris* (hydroxymethyl) aminomethane (Tris) + 6 M guanidine hydrochloride; (4) 100 mM Tris + 6 M guanidine hydrochloride + 0.5 M ethylenediaminetetraacetic acid; or (5) 9 M urea + 4% (m/v) 3-[(3-cholamidopropyl)dimethylammonio]-1-propanesulfonate + 35 mM Tris + 65 mM dithiothreitol, each in the presence of protease inhibitors. Following centrifugation at 14,000 x *g* for 25 min at 4 °C, each supernatant was ultrafiltered using 3 kDa NanoSep centrifugal ultrafilters (Pall, Ann Arbor, MI, USA). In preparation for LC-MS/MS, the ultrafiltrates of the sodium hydroxide extracts were adjusted to pH 2 with trifluoroacetic acid. The ultrafiltrates of extracts containing high levels of chaotropes were dialysed against 0.1 M ammonium bicarbonate in Spectra/Por Float-A-Lyzer units containing 500 Da molecular weight cut-off membranes (Spectrum Laboratories, Inc., Rancho Dominguez, CA, USA), and the dialysates were evaporated to dryness in a CentriVap vacuum centrifuge (LabConco, Kansas City, MI, USA). Retentates from the ultrafiltration step were dissolved in water and proteins were precipitated by the chloroform-methanol-water method of Wessel and Flügge [19]. The isolated proteins were re-suspended in 100 mM Tris containing 6 M urea at pH 7.8 and were reduced with dithiothreitol, alkylated with iodoacetamide and digested with trypsin (1:50 enzyme:substrate ratio).

To potentially extend the range of peptides and proteins extracted from DVA and FDVA, each was also extracted with 0.05 M phosphate buffer + 0.3 M sodium chloride (phosphate-buffered saline, PBS), pH 6.9. Specifically, 1 g of each sample was mixed with 20 mL of PBS and irradiated in an ultrasonication bath (Crest Ultrasonics Corp., Trenton NJ, USA) for 1 h before being mixed in a Mini LabRoller rotator (Labnet International, Inc., Woodbridge, NJ, USA) for 1 h at ambient temperature. After centrifugation at 43,000 x *g* for 15 min at 4 °C, the supernatants were collected and the pellets were re-suspended in 4 mL PBS and then re-centrifuged. The combined supernatants were made up to 25 mL with PBS. Prior to LC-MS/MS analysis, the PBS extracts were heated at 90 °C for 20 min, reduced with 50 mM tris(2-carboxyethyl) phosphine at 56 °C for 45 min, alkylated with 30 mM iodoacetamide at ambient temperature for 30 min, and digested with trypsin (1:50 enzyme:substrate) at 37 °C for 22 h.

Extracts were stored frozen until required for analysis.

### Simulated gastrointestinal digestion

Simulated gastrointestinal digestion of DVA and FDVA was performed using an adaptation of the method of Wang et al. [20]. Specifically, each sample was digested for 2 h at 37 °C with pepsin (1:100 enzyme:substrate) in 0.03 M sodium chloride, pH 2.0. The pH was adjusted to 7.5 with 5 M sodium hydroxide and then pancreatin was added to give an enzyme:substrate ratio of 1:25. After digestion for 2 h at 37 °C, the mixtures were heated for 10 min in a boiling water bath to deactivate enzymes and were then centrifuged at 43,000 x *g* for 15 min at 4 °C. The supernatants were made up to 25 mL with water and were stored at − 80 °C until required for analysis.

### Gel filtration chromatography

Gel filtration chromatography (GFC) was performed on a Knauer HPLC system consisting of a K-1001 pump, S 2600 photo diode array detector, and S 3800 autosampler (Knauer, Berlin, Germany). Samples (50 μL) were injected onto a Yarra SEC-2000 column (300 mm × 7.8 mm i.d, 3 μm; Phenomenex, Torrance, CA, USA), and were eluted at 1 mL/min with 0.05 M phosphate buffer containing 0.3 M sodium chloride, pH 6.9. Column eluent was monitored at 214, 230, 260 and 280 nm. Data were acquired and analysed using Chromgate 3.1 software (Knauer).

### LC-MS/MS analysis

LC-MS/MS was carried out on nanoAdvance HPLCs (Bruker Daltonik GmbH, Bremen, Germany) attached to either an amaZon speed ETD or a maXis impact mass spectrometer (Bruker). A 5 μL sample was loaded on a C18 trap column (5 μm particles, 200 Å pore size; Bruker) at a flow rate of 5 μL/min. The trap column was then switched in-line with the analytical column (C18, 15 cm, 100 μm ID, 3 μm particles, 200 Å pore size; Bruker), which was held in a column oven at 50 °C, and eluted at a flow rate of 800 nL/min with a gradient from 2 to 45% B in either 43 min (for amaZon speed ETD) or 90 min (for maXis impact). Solvent A was 0.1% formic acid, and solvent B was acetonitrile with 0.1% formic acid. On the amaZon speed ETD, four separate runs were performed on pooled extracts of each sample. In three of the runs, collision-induced dissociation (CID) data were acquired for three MS/MS precursor ions per MS survey scan in one of the mass ranges m/z 350–500, 350–650 or 650–1200. In the fourth run, electron-transfer dissociation (ETD) data were acquired. On the maXis impact, each ultrafiltrate, retentate or PBS tryptic digest was separately analysed, with CID data acquired for five MS/MS precursor ions per MS survey scan in the mass range m/z 350–1200 at a sampling rate of 2–5 Hz.

### Peptide identification

MS and MS/MS peak list data were extracted using DataAnalysis v4.1 (Bruker) and imported into ProteinScape v3.1 (Bruker) for protein identification.

Protein identification database searching was performed on an in-house Mascot v2.4 Server (Matrix Science, UK) against two in-house red deer (*Cervus elaphus*) nucleotide databases or the *Bovidae* taxonomy of the National Center for Biotechnology Information (NCBI) non-redundant protein database (12/06/2012). The red deer nucleotide databases consisted of 92,918 and 13,287 expressed sequence tag (EST) contig sequences (containing 63,454,351 and 6,875,407 residues, respectively), which had been annotated by BLAST searches of the NCBI non-redundant protein database. MS/MS search parameters were as follows: enzyme ‘semiTrypsin’ (all extracts) or ‘None’ (all extracts except PBS); two or three missed cleavages allowed; variable modifications carbamidomethyl (C), hydroxylation (KP), deamidation (NQ), oxidation (M) and acetylation (protein N-terminus), grouped with a maximum of three modifications included per search; monoisotopic mass; significance threshold *p* < 0.05; peptide decoy (Mascot) setting selected. Instrument specific parameters were as follows (1) *for maXis impact*: peptide tolerance 50 ppm; MS/MS tolerance 0.5 Da; instrument specificity ESI-QUAD-TOF; (2) *for amaZon speed ETD in CID mode*: peptide tolerance 0.6 Da; MS/MS tolerance 1 Da; instrument specificity ESI-TRAP; and (3) *for amaZon speed ETD in ETD mode*: peptide tolerance 0.6 Da; MS/MS tolerance 1.6 Da; instrument specificity ETD-TRAP.

ProteinScape ProteinExtractor settings were as follows: peptide inclusion threshold 20; peptide acceptance threshold 25; protein acceptance threshold 50, with at least one peptide required with a score 50; for each peptide, accept top hit compound only; in case of MS/MS spectra matching peptides from more than one protein, accept highest scoring peptide (rank 1 peptide).

### ACE activity assay

The activity of ACE in response to four dilutions (undiluted, 1:2, 1:4 and 1:8) of the PBS extract and of the simulated gastrointestinal digest of each sample was measured based on the method of Jimsheena and Gowda [21]. Captopril (1 nM) was used as a positive control for ACE inhibition. A standard curve with known concentrations of hippuric acid (0.5, 1, 2 4, 8, 16 and 32 μM) was used to determine ACE activity. Each sample dilution and control (untreated or captopril-treated) was measured in twelve independent replicates and the data were expressed relative to the untreated control, which was defined as 100% activity.

### In silico analysis of bioactive sequences

A database containing 16,021 entries for peptide sequences having well defined bioactivities was compiled in Microsoft Excel by combining entries from the BIOPEP [17], PeptideDB [22] and APD2 [23] databases along with additional sequences from the scientific literature. Visual Basic for Applications (VBA) macros were then used in Microsoft Excel 2010 to search for three types of matches between peptide sequences in the samples with those in the bioactives database: (1) exact matches of sequences in the samples to bioactive sequences in the database; (2) partial matches of the sequences in the samples to the sequences of the bioactive peptides (i.e. situations in which sequences of the products’ peptides were located within sequences of peptides in the database); and (3) cryptides (i.e. matches of bioactive peptides to portions of the samples’ sequences).

In silico simulated gastric digestion with pepsin was performed on the peptides identified in each sample, using a version of Protein Digestion Simulator [24] which was modified to apply PeptideCutter [25] cleavage specificities. Settings applied in the in silico digestion were cleavage with pepsin (pH > 2), a maximum of three missed cleavages, and a fragment residue count of 2–13.

### Statistical analysis

A random effects model was fitted to the data excluding the untreated ACE control and captopril positive control treatments as they had no simulated gastrointestinal digestion (SGD) treatment. The fixed effects in the model were “DVA” and “SGD” along with their interaction and the random effect was the “run”. The analysis was carried out in R [26].

## Results and discussion

### GFC of PBS extracts and simulated gastrointestinal digests

DVA and FDVA were extracted at pH 6.9 with PBS for GFC analysis. Simulated gastrointestinal digestion was also performed on each sample, and the digests were analysed by GFC under the same conditions as the PBS extracts.

**Fig. 1:**
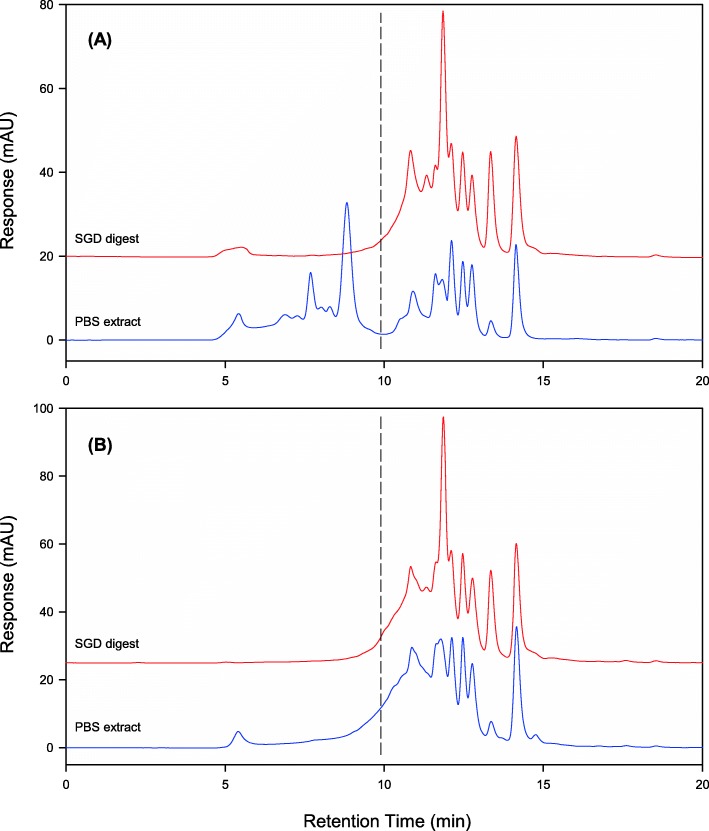
Gel filtration chromatography (GFC) protein profiles of (A) DVA, and (B) FDVA. Detection was performed at 260 nm. In each chromatogram, the trace for the PBS extract (lower) is overlaid by that of the simulated gastrointestinal digest of the same sample (upper). The dashed vertical lines at 9.9 min indicate the retention time at which a 7.5 kDa polypeptide would be expected to elute from the column, based upon calibration of the GFC column with protein standards of known molecular weights. Salt eluted from the column at 11.8 min

Gel filtration chromatograms showing the molecular weight distributions of the PBS extracts and the simulated gastrointestinal digests of DVA and FDVA are presented in Fig. 1. Proteins with molecular weights over 7.5 kDa comprised a significant proportion (53.4%) of the total peak area of the DVA extract, but most of these proteins had been digested in FDVA to peptides of lower molecular weight. In the FDVA chromatogram, only 18.6% of the peak area remained in the over 7.5 kDa molecular weight region. A compensatory increase in the peak area in the mid region of the chromatogram (i.e. the region between the expected elution time of a 7.5 kDa protein and the salt peak) was observed for the fermented sample. This region contributed 46.7% of the signal for FDVA, as compared to only 19.8% for DVA. An appreciable proportion of peptides in both samples eluted from the gel filtration column after the position of the salt peak, amounting to 26.8 and 34.7% for the unfermented and the fermented samples, respectively. This was likely due to separation involving hydrophobic interaction of the peptides with the column packing material, rather than based solely on molecular size.

Simulated gastrointestinal digestion of DVA had a marked effect on its GFC profile. Essentially all of the peaks due to proteins with molecular weights above 7.5 kDa disappeared, replaced by some new peaks in the lower molecular weight region of the chromatogram and by increases in the relative abundance of existing low molecular weight peaks. In contrast, the protein profile of FDVA was virtually unchanged following simulated gastrointestinal digestion. No new peaks were apparent, although there were changes in the relative sizes of some peaks, and the small peak at 5.5 min due to very high molecular weight proteins or aggregates was eliminated. Interestingly, the protein profile of each deer velvet sample was almost indistinguishable following simulated gastrointestinal digestion, as may be seen by comparing the upper trace in Fig. 1A with that in Fig. 1B. This suggested that a pool of similar sized peptides would be expected to be produced following oral consumption of both products.

### LC-MS/MS analysis

The FDVA and DVA samples were extracted using five different extraction systems that together provide efficient extraction of deer velvet proteins. Each of the five extracts was fractionated into free peptide (ultrafiltrate) and protein (retentate) fractions by ultrafiltration with a 3 kDa nominal molecular weight membrane, and the retentates were digested with trypsin for LC-MS/MS analysis.

LC-MS/MS analysis of ultrafiltrates resulted in the identification of over three times as many peptides in the free peptide fractions of the five FDVA buffer extracts as in those of the analogous DVA extracts (1049 and 343 peptides, respectively). This was to be expected based on the increased proportion of sub-7.5 kDa peptides in the fermented sample, as shown by GFC. Conversely, 2.5-fold more peptides were identified in the DVA retentates than in the comparable FDVA retentates after trypsin digestion (785 and 310 peptides, respectively). This was also consistent with the GFC results. Combined, a total of 1644 unique peptide sequences were identified in the ultrafiltrates and retentates of FDVA, compared with 1272 sequences for DVA. The fermentation process used to produce FDVA thus enhanced the overall diversity of peptide sequences present in the soluble portion compared to the unfermented DVA from which it was derived.

PBS extracts of DVA and FDVA were also prepared and were subjected to LC-MS/MS analysis following trypsin digestion. A total of 960 unique peptide sequences were identified in the FDVA digest, as compared to 413 sequences in the DVA. This confirmed that even mild extraction at neutral pH provided a much greater diversity of peptide sequences from FDVA than was obtained from DVA under the same conditions.

To maximise the number of sequences identified in the two deer velvet samples, the identifications resulting from all LC-MS/MS runs of each sample’s ultrafiltrates, retentates and PBS extracts were combined to generate an overall list of unique peptide sequences. This list included sequences of free peptides as well as tryptic fragments derived from the polypeptides and proteins present in each sample. Consistent with the results discussed above, the total number of unique sequences identified in all LC-MS/MS runs of the various extracts and fractions was over 1.5 times higher for FDVA than for DVA (2400 compared to 1561 sequences, respectively). The overall diversity of peptide sequences extractable under both mild and harsh conditions was thus greater from the FDVA sample than from the unfermented DVA.

### In silico prediction of bioactivity

The results of each of the search strategies are separately discussed in the following subsections.

#### Exact matches in the bioactives database

Exact matches to three bioactive peptides were found in the free peptides (ultrafiltrates) of FDVA. These were the closely related sequences LVVYPW, LVVYPWTQ and VVYPWTQ, for which the MS/MS spectra from which they were identified may be seen in Additional file 1. The first is myelopeptide MP-2, which is an immunoregulatory peptide that was first isolated from cultures of porcine bone marrow cells [27]. The other two are extended hemorphin-5 peptides derived from proteolytic degradation of haemoglobin. The hemorphins interact with opioid receptors, have analgesic activity, and are thought to be involved either indirectly (via effects on β-endorphin release) or directly on analgesia and euphoria observed during and after physical exercise. They are also known to be ACE inhibitors, and thus have a positive effect on the cardiovascular system [28]. Each of the three free hemorphins were predicted by in silico gastric digestion to survive proteolysis by pepsin, and in fact to potentially be produced by digestion of longer hemorphin sequences.

In contrast to FDVA, no exact matches to bioactive peptides were found amongst the free peptides of DVA.

#### Matches to partial sequences in the bioactives database

The free peptides in FDVA produced 23 matches to portions of peptides in the database of bioactive peptides, ranging in length from 5 to 22 amino acid residues (Table 1). In comparison, the free peptides of DVA contained nine sequences of between 5 and 17 amino acids in length that partially matched bioactive peptides (Table 1). In both samples most of the partially matched peptides gave hits to varying portions of opioid, antimicrobial and ACE inhibitory peptides derived from bovine haemoglobin. Partial matches to fragments of deer α-globin having wound healing activity [6] were also observed in each sample.

**Table 1:** Partial matches of sample peptides to sequences in the database of bioactive peptides

Sample	Peptide	Matched Name	Activity	Matched Bioactive Sequence*
FDVA	VVYPWTQR	LVV-hemorphin-6	Opioid/ACE inhibitor	L**VVYPWTQR**
	FLSFPTTK	Bovine haemoglobin peptide	Antimicrobial	**FLSFPTTK**TYFPHFDLSHGSAQVKGHGAK
	FLSFPTTKTYFPH			**FLSFPTTKTYFPH**FDLSHGSAQVKGHGAK
	SFPTTK			FL**SFPTTK**TYFPHFDLSHGSAQVKGHGAK
	SFPTTKTYFPH			FL**SFPTTKTYFPH**FDLSHGSAQVKGHGAK
	SFPTTKTYFPHFDLSHGSAQ			FL**SFPTTKTYFPHFDLSHGSAQ**VKGHGAK
	SFPTTKTYFPHFDLSHGSAQVK			FL**SFPTTKTYFPHFDLSHGSAQVK**GHGAK
	TYFPH			FLSFPTTK**TYFPH**FDLSHGSAQVKGHGAK
	TYFPHFDL			FLSFPTTK**TYFPHFDL**SHGSAQVKGHGAK
	TYFPHFDLSH			FLSFPTTK**TYFPHFDLSH**GSAQVKGHGAK
	TYFPHFDLSHG			FLSFPTTK**TYFPHFDLSHG**SAQVKGHGAK
	TYFPHFDLSHGS			FLSFPTTK**TYFPHFDLSHGS**AQVKGHGAK
	TYFPHFDLSHGSA			FLSFPTTK**TYFPHFDLSHGSA**QVKGHGAK
	TYFPHFDLSHGSAQ			FLSFPTTK**TYFPHFDLSHGSAQ**VKGHGAK
	TYFPHFDLSHGSAQVK			FLSFPTTK**TYFPHFDLSHGSAQVK**GHGAK
	SRAGLQFPVGRVH	Buforin II	Antimicrobial	TRS**SRAGLQFPVGRVH**RLLRK
	QVSLNSGY	ACE inhibitor	ACE inhibitor	**QVSLNSGY**Y
	AAWGKVGGNAPAF	Wound healing peptide	Wound healing/ immunomodulatory	VLSAADKSNVK**AAWGKVGGNAPAF**GAEALLRM
	AAWGKVGGNAPAFGAE			VLSAADKSNVK**AAWGKVGGNAPAFGAE**ALLRM
DVA	SFPTTKTYFPHFDLSHG	Bovine haemoglobin peptide	Antimicrobial	FL**SFPTTKTYFPHFDLSHG**SAQVKGHGAK
	PTTKTYFPHFDLSH			FLSF**PTTKTYFPHFDLSH**GSAQVKGHGAK
	PTTKTYFPHFDLSHG			FLSF**PTTKTYFPHFDLSHG**SAQVKGHGAK
	TKTYFPHFDLSH			FLSFPT**TKTYFPHFDLSH**GSAQVKGHGAK
	TKTYFPHFDLSHG			FLSFPT**TKTYFPHFDLSHG**SAQVKGHGAK
	TYFPHFDLSH			FLSFPTTK**TYFPHFDLSH**GSAQVKGHGAK
	TYFPHFDLSHG			FLSFPTTK**TYFPHFDLSHG**SAQVKGHGAK
	VLSAADKSNVK	Wound healing peptide	Wound healing/ immunomodulatory	**VLSAADKSNVK**AAWGKVGGNAPAFGAEALLRM

*The portions of the bioactive sequences that were matched by the peptides in FDVA and DVA are shown in bold

#### Cryptides

Large numbers of short bioactive peptides sequences gave partial matches to peptide sequences identified in FDVA and DVA (Table 2). For the free peptides (ultrafiltrates), the number of individual matched bioactive peptides, and also the total number of hits to those sequences, was greater in FDVA’s peptides than in those of DVA. When the sequences that were identified from tryptic digests of intact proteins (retentates) of the two samples were included, it was DVA that had the greater number of matches to individual bioactive peptides. However, despite matching a lower number of individual bioactive peptides, FDVA still had the greater total number of hits to matched bioactive sequences when all its identified peptides were considered.

**Table 2:** Cryptides identified in sample peptides

Sample	Number of Individual Cryptides	Average Length of Cryptides	Total No. of Cryptides Hits
*Free peptides:*			
FDVA	222	2.75 ± 1.41	9660
DVA	156	2.37 ± 0.67	2477
*All peptides:*			
FDVA	308	2.89 ± 1.42	26,085
DVA	347	2.98 ± 1.46	19,053

Data are presented for the free peptide fractions (i.e. ultrafiltrates), and for the overall results of all extracts and fractions combined in extracts of FDVA and DVA. The cryptide lengths data are means ± standard deviations

The average length of cryptides was slightly greater for FDVA than DVA in the free peptides fractions, and the variance was also greater for the former (Table 2). This was due to the identification of 17 bioactive peptides containing between 5 and 13 amino acid residues that partially matched sequences identified amongst FDVA’s free peptides. In contrast, the longest bioactive peptides that gave partial matches to DAV’s free peptides only contained five amino acid residues, and there were only three such matching pentapeptides. When all the identified peptides of each sample were included, however, the average length of the matched bioactive sequences and the variance was similar for both samples. This no doubt reflects the common starting pool of proteins in the samples, given that FDVA was produced by fermentation of DVA. In all cases, the average length of matched bioactive sequences was less than three amino acid residues. Thus, the bioactive peptides that produced matches were predominantly dipeptides and tripeptides. In both samples, the bioactive peptide that was most frequently matched was glycine-proline (GP). When GP was matched in a peptide’s sequence, on average it occurred twice within that sequence. Most other matched bioactive sequences occurred only a single time within the containing sample peptide. In silico digestion with pepsin predicted that all of the short bioactive sequences detected within the peptides of FDVA and DVA could be produced during gastric digestion. And, with three missed cleavages permitted during the in silico digestion, the bioactive fragments up to nine residues in length were also all predicted.

In the database of bioactive peptides used in this study, each peptide is categorised according to its reported biological activity. Some peptides have multiple activities, and thus occur in the database multiple times assigned to different categories.

**Fig. 2:**
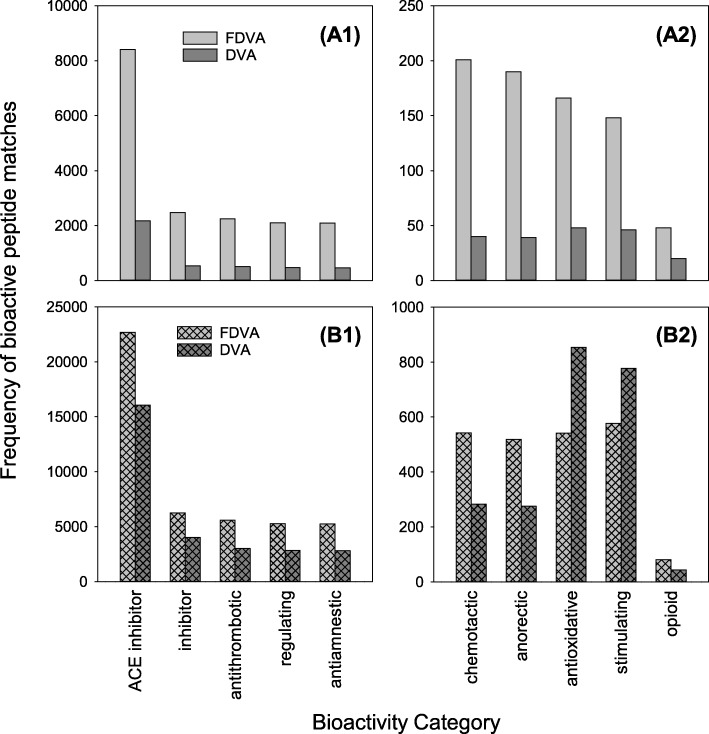
Frequencies of bioactive peptide matches to portions of peptide sequences identified by in silico analysis in DVA and FDVA. The frequencies are plotted by: (A1 and A2) bioactivity category for free peptides (i.e. ultrafiltrates); and (B1 and B2) all peptides (i.e. ultrafiltrates and retentates combined)

The frequencies with which cryptides, categorised according to their biological activities, had hits within sequences of the samples’ peptides are graphically displayed in Fig. 2. Amongst the free peptides, the greater number of matches to each category of bioactive peptides was, without exception, found in FDVA (Fig. 2A). For most categories, the number of matches in FDVA was about four times that of DVA. Thus, in the most bioavailable fraction of the two samples, FDVA had the greatest potential to generate short bioactive peptides. When all identified peptide sequences (including those from intact proteins) were considered, FDVA still had considerably more matches than DVA for many bioactivity categories (Fig. 2B). However, for ‘anti-oxidative’ and ‘stimulating’ activities, greater numbers of hits were instead found in DVA. The cryptides responsible for hits to each bioactivity category are discussed below.

By far the most commonly matched biological activity was ‘ACE inhibitor’. This was due to the fact that many dipeptides and tripeptides that contain one or more hydrophobic amino acids – e.g. GP, PG, AG – were matched with high frequency in the samples’ peptides, and these short peptides are known inhibitors of ACE [29].

Another very common activity observed was ‘inhibitor’, which includes inhibitors of other enzymes. Most of the matches in this category were to inhibitors of dipeptidyl-aminopeptidase IV (DPP4). DPP4 is an enzyme expressed on the surface of most cells that plays a major role in glucose metabolism [30]. This makes inhibitors of DPP4 of great interest for managing type 2 diabetes, especially since they have a negligible risk of inducing hypoglycaemia [31].

Antithrombotic activity was the next most commonly matched bioactivity. Thrombosis is a pathological condition involving blood clot formation. Most antithrombotic peptides of food origin that have been identified are derived from enzymatic hydrolysis of κ-casein in milk [32].

A significant number of hits to ‘regulating’ cryptides was observed. These were almost exclusively due to matches to the sequences GP, PG and PGP, which have been shown to be involved in homeostasis of gastric mucosa [33], and which are considered promising for prevention and treatment of stomach and duodenal ulcers .

The same proline-containing dipeptides and tripeptide were also mainly responsible for the matches to ‘antiamnestic’ activity, owing to their ability to inhibit prolyl oligopeptidase (POP) [33]. POP is an enzyme that degrades proline-containing neuropeptides involved in memory and learning, such as vasopressin, substance P and thyrotropin-releasing hormone. Inhibitors of POP are therefore regarded as potential therapeutics for treatment for dysfunction of the memory system (e.g. Alzheimer’s disease) and for the improvement of general cognitive behaviour in the elderly [34].

The matches to ‘chemotactic’ activity were due to hits to the peptides PGP and VGAPG, which both attract neutrophils in blood [35, 36].

The PGP sequence was also almost the sole cause of the matches to the ‘anorectic’ (appetite suppression) bioactivity. PGP has been shown to inhibit insulin secretion [37].

A wider range of di-, tri- and tetrapeptides were responsible for the matches to ‘antioxidative’ activity. The antioxidant activity of bioactive peptides can be attributed to their radical scavenging, inhibition of lipid peroxidation and metal ion chelation properties. The well-established reverse relationship between antioxidant intake and various diseases has caused great research interest in natural antioxidant peptides derived from food [37].

The assignment of ‘stimulating’ activity was mostly due to matches to a number of hydrophobic branched-chain amino acid containing dipeptides (VL, LL, LV, IL, LI, IV and II) that stimulate glucose uptake in skeletal muscles, resulting in increased skeletal muscle glycogen contents [38]. The other hits with ‘stimulating’ activity were to dipeptides and tripeptides (e.g. SE, VPL, EEE and SSS), which have been shown to influence the release of various vasoactive substances by endothelial cells and have been linked to antiatherogenic properties of soy protein and casein hydrolysates [39].

The ‘opioid’ activity was due to hits to hemorphin-4 and hemorphin-5, as well as to tripeptides PLG (gliadin 1 exorphin) and GLF and the pentapeptide TSKYR (neokyotorphin). The analgesic and ACE inhibitory properties of hemorphins were discussed above. Neokyotorphin also exhibits strong analgesic activity [40].

Taken together, these results demonstrated that both fermented and unfermented deer velvet have considerable potential to generate short bioactive peptides expressing a variety of biological activities. Realisation of that potential obviously requires proteolytic degradation to release the bioactive peptides. In silico digestion with pepsin confirmed that generation of all the short peptides discussed above could be expected to occur following oral consumption.

### In vitro ACE inhibitory activity

Given the presence of a number of ACE inhibitors detected within the free peptides of FDVA, together with the considerable number of potential ACE inhibitor peptides encoded within the peptides and proteins of both samples, it was of interest to investigate whether extracts of the two samples would in fact demonstrate significant inhibition of ACE. In vitro assessment of ACE activity was performed both before and after simulated gastrointestinal digestion, to determine whether proteolysis by gastrointestinal enzymes would release additional ACE inhibitors as predicted by in silico digestion. In an exploratory analysis of the ACE activity results, the different concentrations of FDVA and DVA examined had little effect on ACE activity and consequently those data were combined for the final statistical analysis. Further work would be required to fully investigate dose effects of the deer velvet extracts on ACE inhibition.

ACE activity was significantly lower for the positive control (1 nM captopril), and for FDVA and DVA, each compared to untreated controls (*P* < 0.001) (Table 3). Prior to simulated gastrointestinal digestion, the inhibition observed for FDVA was greater than for DVA (*P* < 0.01). This is consistent with the detection of matches to free ACE inhibitors in FDVA but not in DVA. It is also likely that the fermentation used to produce FDVA would have generated other short peptides (di-, tri- and tetrapeptides) that went undetected, since the LC-MS/MS peptide identification method required at least five amino acid residues to produce good matches during searching of the protein database. Based on the in silico analysis, such short peptides would be expected to include ACE inhibitors.

**Table 3:** ACE activity of PBS extracts before and after simulated gastrointestinal digestion (SGD)

Sample	SGD	ACE activity (%)
Untreated control	–	100 ± 5.23^*a*^
Captopril	–	73.99 ± 5.23^*b*^
FDVA	no	16.36 ± 5.52^*c*^
	yes	17.57 ± 5.52^*c*^
DVA	no	41.55 ± 5.52^*d*^
	yes	17.57 ± 5.52^*c*^

Data presented are means ± standard errors of 12 replicates. Captopril (1 nM) was included as a positive control treatment. Values with different subscripts differ significantly (*P* < 0.001 for comparisons *bc* and *bd,* and for all comparisons with the untreated control; *P* < 0.01 for comparison *cd*)

Following simulated gastrointestinal digestion, the effect of FDVA on ACE activity was unchanged (Table 3). Thus, it would seem that the gastrointestinal enzymes were neither able to markedly increase the pool of ACE inhibitory peptides, nor to degrade them and thus reduce ACE inhibition. This suggested that the proteases present during the fermentation process had degraded the velvet proteins in a fashion similar to pepsin to release as many of the potential ACE inhibitors as possible, and that any antihypertensive benefits of FDVA that would be conferred by its ACE inhibitory activity would not be affected by, or dependent upon, proteolytic degradation following oral ingestion.

In contrast, simulated gastrointestinal digestion of DVA significantly enhanced ACE inhibition (*P* < 0.01) to the level of FDVA. Thus, as had been predicted by the in silico digestion analysis, the level of ACE inhibitors in DVA was increased by pepsin digestion. Like FDVA, DVA would be expected to convey positive antihypertensive effects following oral consumption.

## Conclusions

In conclusion, we have for the first time in this study performed a large scale peptidomic analysis of DVA and combined this with an in silico and in vitro analysis of bioactivity. The in silico analysis revealed that DVA contains a large number of potential bioactive peptide sequences, and that this was increased by fermentation. Fermentation also released three bioactive hemorphin peptides, specifically LVVYPW, LVVYPWTQ and VVYPWTQ, which wouldn’t require proteolytic processing during oral consumption to be made bioavailable. It probably also produced very short bioactive peptides that were not identified by the LC-MS/MS method employed. The in silico analysis predicted that strong inhibition of ACE should be exhibited by both fermented and unfermented DVA. This activity was confirmed in vitro, and it was further demonstrated that simulated gastrointestinal digestion strongly enhanced the level of ACE inhibition of the standard DVA as inferred from the in silico analysis. In contrast, the ACE inhibition of FDVA was not further enhanced by simulated gastrointestinal digestion. Apparently the fermentation process had already substantially digested the proteins present in DVA and further digestion by gastrointestinal enzymes did not markedly increase the level of protein hydrolysis. This conclusion was supported by the gel filtration results, which showed that simulated gastrointestinal digestion had only a minimal effect on the molecular weight profile of FDVA while that of standard DVA was markedly changed.

The results have demonstrated the power of in silico analysis for predicting the bioactivity of complex mixtures of peptides and proteins from functional food sources and have shown that, in addition to its many other beneficial properties, DVA would be expected to have beneficial antihypertensive effects derived from its ability to inhibit ACE. Further work involving in vivo studies would be required to provide definitive confirmation of this deduction.

## Supplementary information

**Additional file 1.** ACE inhibitory peptides in standard and fermented deer velvet: an in silico and in vitro investigation. Contents: MS/MS spectra of three bioactive peptides identified in the FDVA sample.

## Data Availability

The datasets used and/or analysed during the current study are available from the corresponding author on reasonable request.

## Abbreviations

ACE: angiotensin-converting enzyme
CID: collision-induced dissociation
DPP4: dipeptidyl-aminopeptidase IV
DVA: deer velvet antler
EST: expressed sequence tag
ETD: electron-transfer dissociation
FDVA: fermented deer velvet antler
GFC: gel filtration chromatography
LC-MS/MS: liquid chromatography-tandem mass spectrometry
MS: mass spectrometry
MS/MS: tandem mass spectrometry
NCBI: National Center for Biotechnology Information
PBS: phosphate-buffered saline
POP: prolyl oligopeptidase
SGD: simulated gastrointestinal digestion
Tris: *tris* (hydroxymethyl)aminomethane
VBA: Visual Basic for Applications
