# Supplementary material for: ACE inhibitory peptides in standard and fermented deer velvet: an in silico and in vitro investigation
Source: BMC Complement Altern Med. 2019 Dec 5;19:350. doi: 10.1186/s12906-019-2758-3 (PMC6896680; doi:10.1186/s12906-019-2758-3)
Supplement: Supplementary file 1 — Additional file 1. ACE inhibitory peptides in standard and fermented deer velvet: an in silico and in vitro investigation. Contents: MS/MS spectra of three bioactive peptides identified in the FDVA sample. [file 12906_2019_2758_MOESM1_ESM.docx]

**ACE inhibitory peptides in standard and fermented deer velvet: an *in silico* and *in vitro* investigation – Supplementary Information**

Figures S1–S3 show MS/MS spectra of the three bioactive peptides (LVVYPW, LVVYPWTQ and VVYPWTQ) that were identified in the ultrafiltrates of FDVA (see section 3.3.1 in the manuscript). The spectra were acquired on the maXis impact Q-TOF mass spectrometer, and the peak assignments are as displayed by ProteinScape. Each of the spectra exhibits the expected intense *y*-ion signal for fragments terminating in proline.


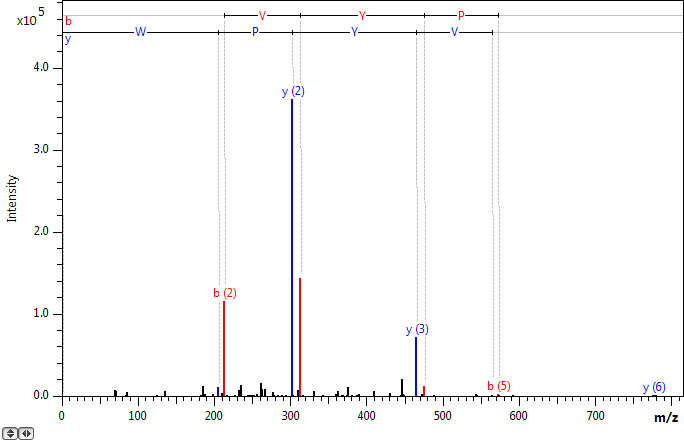


**Figure S1.** The MS/MS spectrum of LVVYPW. The singly charged precursor ion was observed at m/z 776.4334, equivalent to a mass error of 0.96 ppm. The Mascot score for the peptide identification was 27.7.


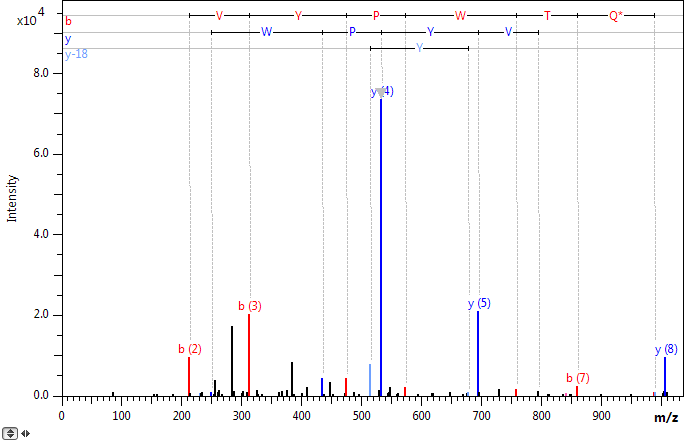


**Figure S2.** The MS/MS spectrum of LVVYPWTQ, showing a deamidation modification of Q. The singly charged precursor ion was observed at m/z 1006.5196, equivalent to a mass error of ‑4.74 ppm. The Mascot score for the peptide identification was 33.4.


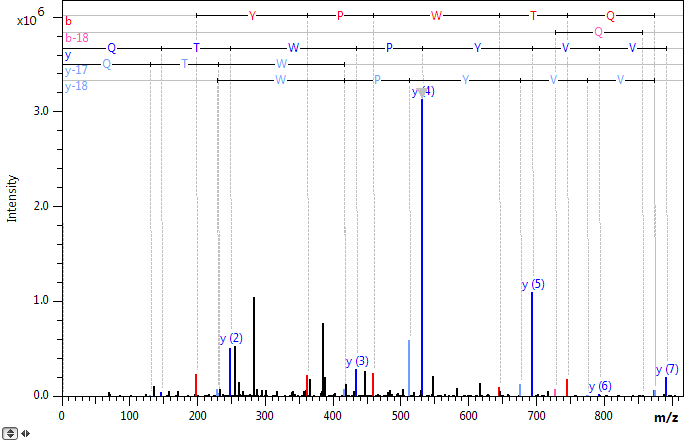


**Figure S3.** The MS/MS spectrum of VVYPWTQ. The singly charged precursor ion was observed at m/z 892.4544, equivalent to a mass error of -2.14 ppm. The Mascot score for the peptide identification was 35.5.
